# Development and validation of a novel nomogram for predicting lateral lymph node metastasis in medullary thyroid carcinoma

**DOI:** 10.3389/fendo.2025.1648529

**Published:** 2025-09-19

**Authors:** KeJie Yu, XianJiang Wu, WeiDong Zhang

**Affiliations:** Department of Thyroid Surgery, Ningbo No.2 Hospital, Ningbo, Zhejiang, China

**Keywords:** medullary thyroid carcinoma, lateral lymph node dissection, lateral lymph node metastasis, nomogram, risk stratification

## Abstract

**Background:**

Medullary thyroid carcinoma (MTC) frequently presents with lateral lymph node metastasis (LLNM), a critical determinant of postoperative recurrence. While surgery remains the cornerstone of MTC treatment, the indications for lateral lymph node dissection (LLND) remain contentious. This study aimed to develop and validate a predictive nomogram for assessing LLNM risk in patients with MTC.

**Methods:**

We retrospectively analyzed 87 treatment-naïve MTC patients who underwent primary surgical resection at our institution. Univariate and multivariate logistic regression analyses were performed to identify independent risk factors for LLNM. A nomogram was constructed and internally validated, with its clinical utility evaluated through discrimination, calibration, and decision curve analyses.

**Results:**

Univariate analysis identified multifocality, intrathyroidal lymphovascular invasion (IT-LVI), extrathyroidal extension (ETE), central lymph node metastasis (CLNM), maximum tumor diameter (MTD), serum calcitonin (Ctn), and carcinoembryonic antigen (CEA) as significantly associated with LLNM (P < 0.05). Multivariate logistic regression analysis revealed ETE (OR = 14.37; 95% CI: 2.11–100.24; P = 0.007), CLNM (OR = 4.97; 95% CI: 1.06–23.26; P = 0.042), and natural log-transformed Ctn (Ln_Ctn) (OR = 2.72; 95% CI: 1.49–4.99; P<0.001) as independent predictors. The resulting nomogram demonstrated excellent discriminative ability (AUC = 0.941), good calibration, and strong clinical utility.

**Conclusion:**

We developed a novel nomogram incorporating ETE, CLNM, and Ln_Ctn to accurately estimate LLNM probability in MTC patients. This predictive model significantly improves risk stratification, provides valuable guidance for surgical decision-making regarding LLND, and supports personalized surgical planning.

## 1 Introduction

Medullary thyroid carcinoma (MTC) is a rare neuroendocrine malignancy originating from parafollicular C-cells of the thyroid, accounting for 1–2% of all thyroid cancers (1), but 8–14% of thyroid cancer-related deaths (2, 3). Unlike differentiated thyroid carcinomas, MTC is characterized by early and aggressive lymphatic spread, with cervical lymph node metastases — particularly to the lateral lymph node metastasis (LLNM) — serving as crucial prognostic indicators (4). At initial diagnosis, 40–50% of patients present with either central lymph node metastasis (CLNM) or LLNM (5, 6), both of which are strongly associated with locoregional recurrence and poorer survival outcomes (7).

Surgical resection remains the only potentially curative treatment for MTC (8). However, the optimal extent of lymph node dissection, particularly regarding prophylactic lateral lymph node dissection (LLND), remains debated across guidelines. While the American Thyroid Association (ATA) recommends total thyroidectomy with central compartment dissection (1), the optimal extent of initial nodal dissection remains debated, particularly regarding lateral compartment clearance. National Comprehensive Cancer Network (NCCN) suggests that central dissection may be omitted for tumors ≤1 cm without additional risk factors (9). Furthermore, elevated preoperative serum calcitonin (Ctn) has been proposed as a surrogate marker for metastatic disease, with values >20 pg/mL and >200 pg/mL associated with ipsilateral and contralateral LLNM, respectively (10, 11).

In clinical practice, surgical decisions are often guided by intraoperative judgment or surgeon experience, which can result in either inadequate resection or unnecessary morbidity. Incomplete lymphadenectomy increases recurrence risk, while overtreatment elevates complications such as recurrent laryngeal nerve injury and permanent hypoparathyroidism (12). Therefore, identifying reliable predictors of LLNM and developing validated, quantitative models are imperative for individualized surgical decision-making (13).

Despite ongoing research into MTC’s molecular characteristics and the emergence of targeted therapies, surgical resection remains the primary curative approach. Yet, international consensus on LLNM predictors is lacking. A meta-analysis of 26 studies found significant heterogeneity in reported risk factors for LLNM, highlighting the need for more consistent and validated tools (14). Our study addresses this gap by systematically evaluating clinicopathological features of MTC and constructing a predictive nomogram to guide personalized LLND decision-making and optimize patient outcomes.

## 2 Materials and methods

### 2.1 Patients and data collection

This retrospective study included 87 consecutive patients with pathologically confirmed MTC who underwent initial thyroid surgery at Ningbo No. 2 Hospital between 2016 and 2024. All enrolled MTC patients underwent central and lateral lymph node dissection (LND). Therapeutic LND was performed for patients with radiologically or cytologically confirmed nodal metastasis, while prophylactic LND was conducted for those without preoperative evidence of metastasis. Notably, both approaches followed identical anatomical dissection boundaries. The study aimed to analyze clinical and pathological features to assess risk factors for LLNM. Inclusion criteria were as follows: (1) histopathological confirmation of MTC after surgery; (2) complete preoperative clinical and laboratory data; (3) availability of thyroid color Doppler ultrasound (US) and neck computed tomography (CT) imaging; (4) No prior treatment for thyroid malignancy; (5) underwent total thyroidectomy.

Clinical data were extracted from the hospital’s electronic medical record system and the Ningbo Resident Health Big Data Platform. Collected variables included: (1) demographic and clinical characteristics (preoperative assessment): gender, age, body mass index (BMI); (2) tumor-related features: maximum tumor diameter (MTD, preoperative imaging by US/CT), extrathyroidal extension (ETE, preoperative imaging by US/CT); (3) Serological markers (preoperative laboratory testing): serum Ctn, carcinoembryonic antigen (CEA); (4) Pathological findings (postoperative histopathology): intrathyroidal lymphovascular invasion (IT-LVI), CLNM, and LLNM.

### 2.2 Construction and validation of the prediction model

Risk factors associated with LLNM were first screened through univariate logistic regression analysis. Variables with statistical significance were then included in multivariate logistic regression models to identify independent predictors, adjusting for potential confounding factors. Based on the multivariate results, a predictive nomogram was constructed to estimate the probability of LLNM. Internal validation was performed using 1,000 bootstrap resamples. Calibration of the model was assessed via calibration plots. Discrimination was evaluated by calculating the area under the receiver operating characteristic (ROC) curve (AUC). Clinical utility was assessed through decision curve analysis (DCA), which estimated net benefit across a range of threshold probabilities.

### 2.3 Statistical analysis

All statistical analyses were conducted using SPSS version 27.0 (IBM Corp., Armonk, NY, USA) and R version 4.4.3 (R Foundation for Statistical Computing, Vienna, Austria). Univariate analyses employed: χ² tests for categorical variables; independent t-tests for normally-distributed continuous variables; and Mann-Whitney U tests for non-normally distributed variables. Variables demonstrating statistical significance (P<0.05) in univariate analysis were entered into multivariate logistic regression models, with outcomes reported as odds ratios (ORs) with 95% confidence intervals (CIs). A two-sided P-value <0.05 defined statistical significance. Forest plots and ROC curves were generated using GraphPad Prism version 10.1.2 (GraphPad Software, San Diego, CA, USA). Nomogram construction, waterfall plots, calibration plots, and DCA were all performed in R, with internal validation conducted through 1,000 bootstrap replicates.

To evaluate predictive performance, we conducted ROC curve analysis using DeLong’s method to calculate the AUC with its 95% confidence interval. The optimal cutoff was determined by maximizing Youden’s index (sensitivity + specificity - 1), with the corresponding sensitivity and specificity values reported. For the nomogram, no single optimal cutoff exists; instead, we used the 50% predicted probability and its corresponding score as a clinical reference point. Additionally, model calibration was assessed using the Brier score, while clinical utility was evaluated through decision curve analysis (DCA).

## 3 Results

This retrospective analysis included 87 consecutive patients with MTC, among whom 27 (31.0%) had pathologically confirmed LLNM.

### 3.1 Analysis of risk factors for LLNM

**Figure 1:**
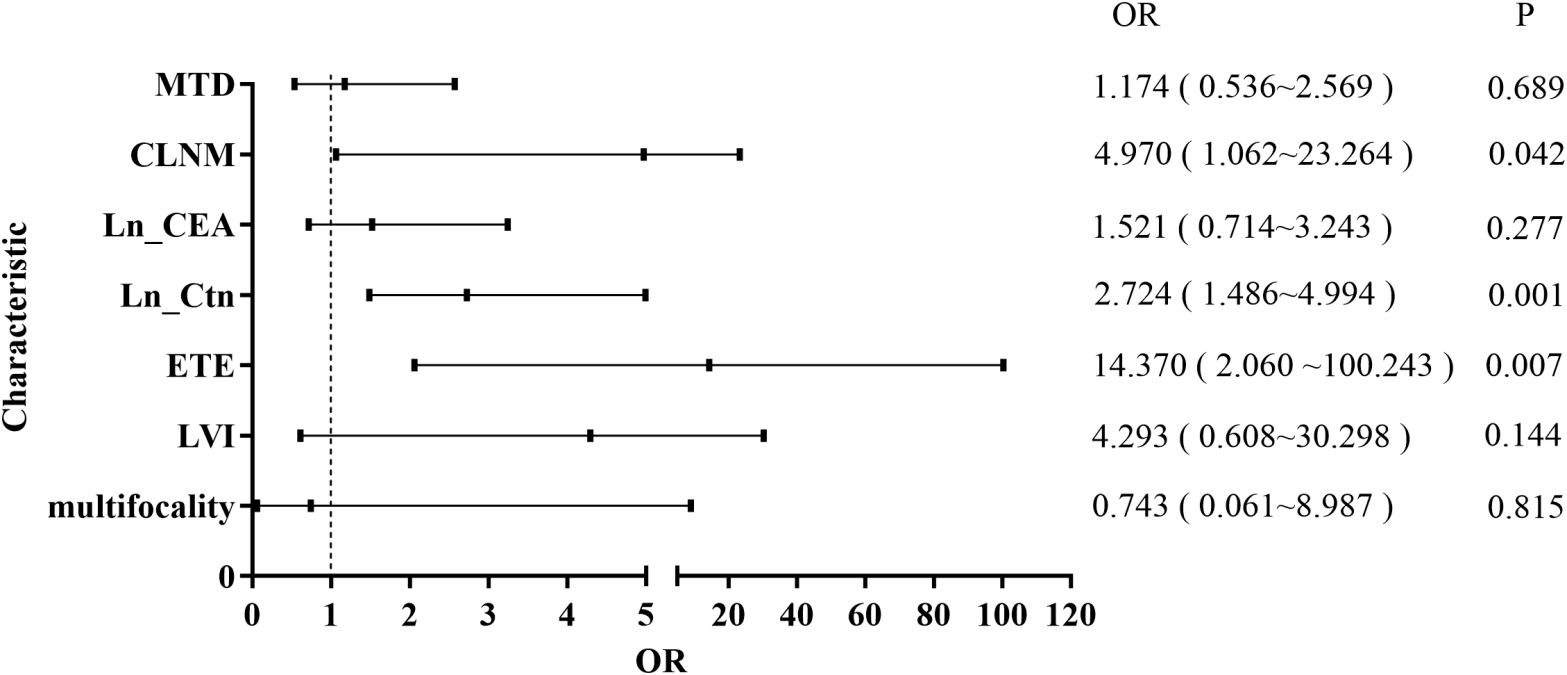
Forest plot of multivariate logistic regression analysis for risk factors associated with LLNM.

To identify risk factors associated with LLNM, patients were categorized into LLNM-positive (n = 27) and LLNM-negative (n = 60) groups. Univariate analysis revealed that multifocality (P = 0.004), IT-LVI (P < 0.001), ETE (P < 0.001), CLNM (P < 0.001), MTD (P < 0.001), Ctn (P < 0.001), and CEA (P < 0.001) were significantly associated with LLNM (**Table 1**). Subsequent multivariate logistic regression analysis identified ETE (OR = 14.37; 95% CI: 2.11–100.24; P = 0.007), CLNM (OR = 4.97; 95% CI: 1.06–23.26; P = 0.042), and Ln-transformed Ctn (Ln_Ctn) (OR = 2.72; 95% CI: 1.49–4.99; P < 0.001) as independent predictors of LLNM (**Figure 1**). Log transformation of Ctn values was applied to reduce skewness and improve model robustness. These findings suggest that MTC patients with ETE, elevated serum Ctn levels, and CLNM are at significantly increased risk for LLNM.

**Table 1:** Univariate analysis of the clinical and pathological factors associated with LLNM.

Characteristic	Number of patients	LLNM		P-value
	N=87	Positive (n=27)	Negative (n=60)	
Gender				
Male	35	14 (40%)	21 (60)	0.138
Female	52	13 (25%)	39 (75%)	
Multifocality				
Yes	11	8 (72.73%)	3 (27.27%)	0.004
No	76	19 (25%)	57 (75%)	
IT-LVI				
Yes	19	15 (78.95%)	4 (21.05%)	< 0.001
No	68	12 (17.65%)	56 (82.35%)	
ETE				
Yes	21	16 (76.19%)	5 (23.80%)	< 0.001
No	66	11 (16.67%)	55 (83.33%)	
CLNM				
Yes	39	23 (58.97%)	16 (41.03%)	< 0.001
No	48	4 (8.33%)	44 (91.67%)	
Age (years)	53.09 ± 12.14	54.67 ± 12.72	52.38 ± 11.91	0.420
(mean ± SD)				
BMI (kg/m^2^)	23.88 ± 3.18	23.33 ± 3.14	24.13 ± 3.20	0.281
(mean ± SD)				
MTD (cm)	1.3 (0.7-2.4)	2.0 (1.3-4.0)	1.10 (0.6-1.96)	< 0.001
(median, IQR)				
Ctn (pg/mL)	230.4 (14.4-1331.5)	2000 (433.9-2000)	124.70(7.19-420.93)	< 0.001
(median, IQR)				
CEA(ng/mL)	13.89 (2.51-46.2)	49.56 (17.16-172.31)	6.01 (1.93-21.06)	< 0.001
(median, IQR)				

CLNM, Central lymph node metastasis; IT-LVI, Intrathyroidal lymphovascular invasion; ETE, Extrathyroidal extension; BMI, Body mass index; MTD, Maximal tumor diameter; Ctn, Calcitonin; CEA, Carcinoembryonic antigen.

### 3.2 Development and validation of the predictive nomogram

**Figure 2:**
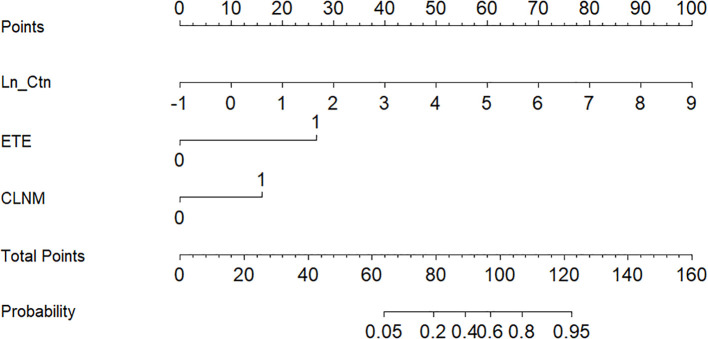
Nomogram for predicting the risk of LLNM in MTC.

Based on the independent predictors identified in the multivariate analysis, a nomogram was developed to estimate the probability of LLNM in MTC patients (**Figure 2**). The scoring algorithm assigned 27 points for ETE, 16 points for CLNM, and 10 points per unit increase in Ln_Ctn. An optimal cutoff of 93 total points corresponded to a 50% predicted probability of LLNM.

**Figure 3:**
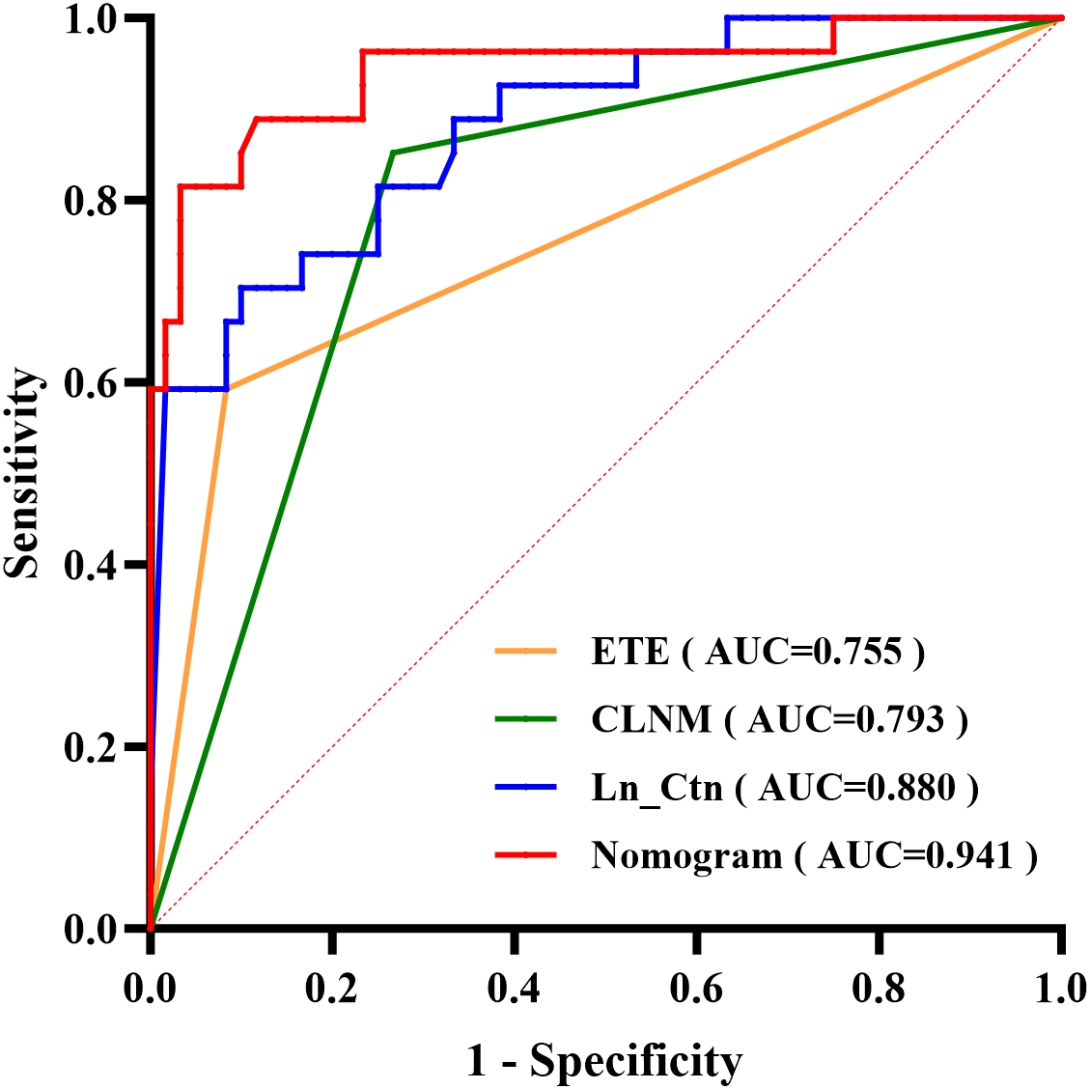
ROC curves comparing the predictive performance of the nomogram versus three independent risk factors.

**Figure 4:**
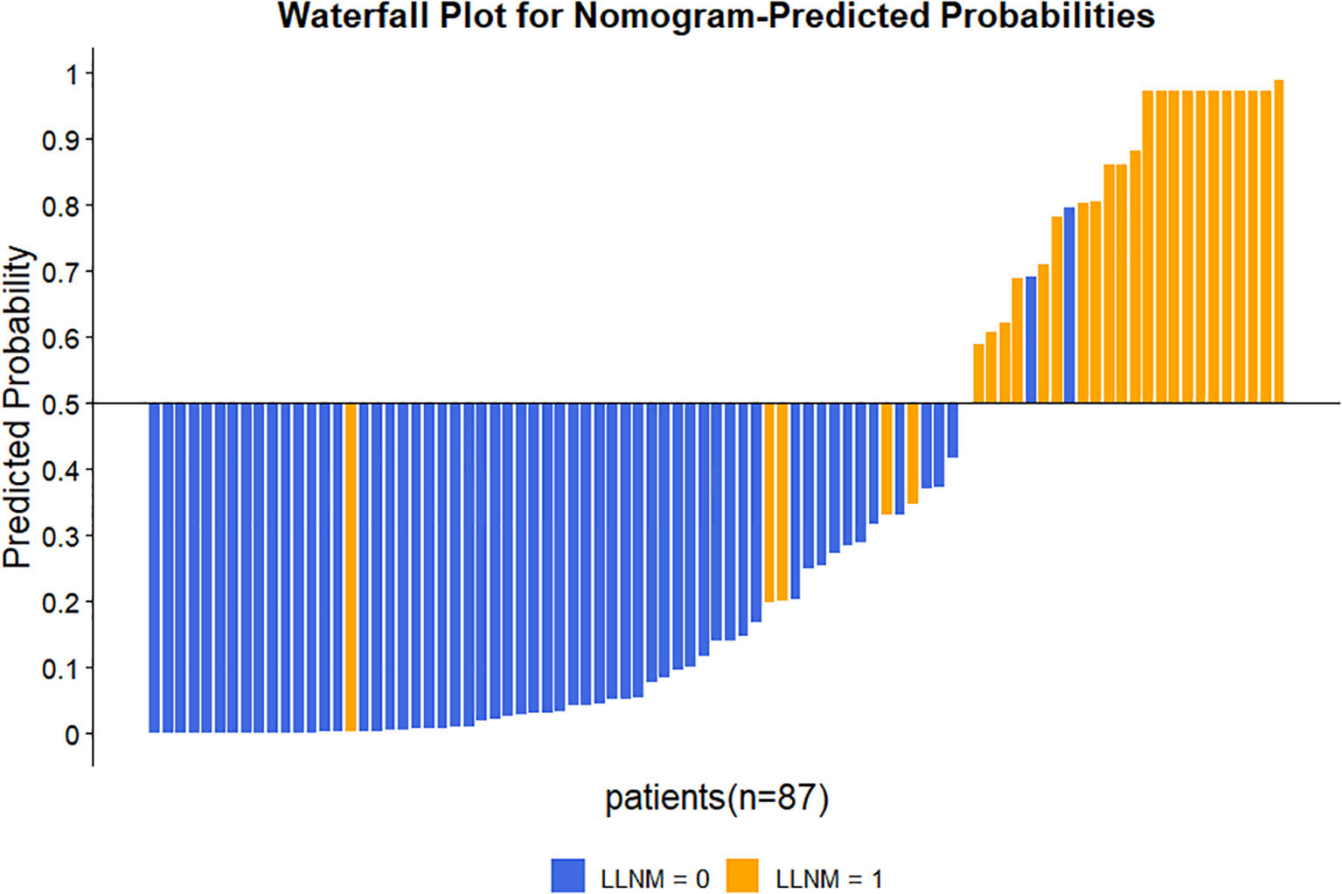
Waterfall plot of predicted probability distribution for LLNM in MTC.

The nomogram’s discriminative ability was evaluated using receiver operating characteristic (ROC) curve analysis. The model demonstrated excellent performance, with an area under the curve (AUC) of 0.941 (95% CI: 0.881–1.000), yielding a sensitivity of 81.5% and specificity of 96.7% (**Figure 3**). For comparison, the AUC values of the individual predictors were 0.880 (95% CI: 0.803–0.957) for Ctn, 0.755 (95% CI: 0.632–0.877) for ETE, and 0.793 (95% CI: 0.690–0.895) for CLNM. At the 50% probability threshold, the waterfall plot (**Figure 4**) illustrates the predicted LLNM probabilities across all 87 patients. Both the ROC analysis and probability distribution visualization confirmed the nomogram’s superior predictive accuracy for LLNM compared to each individual risk factor.

**Figure 5:**
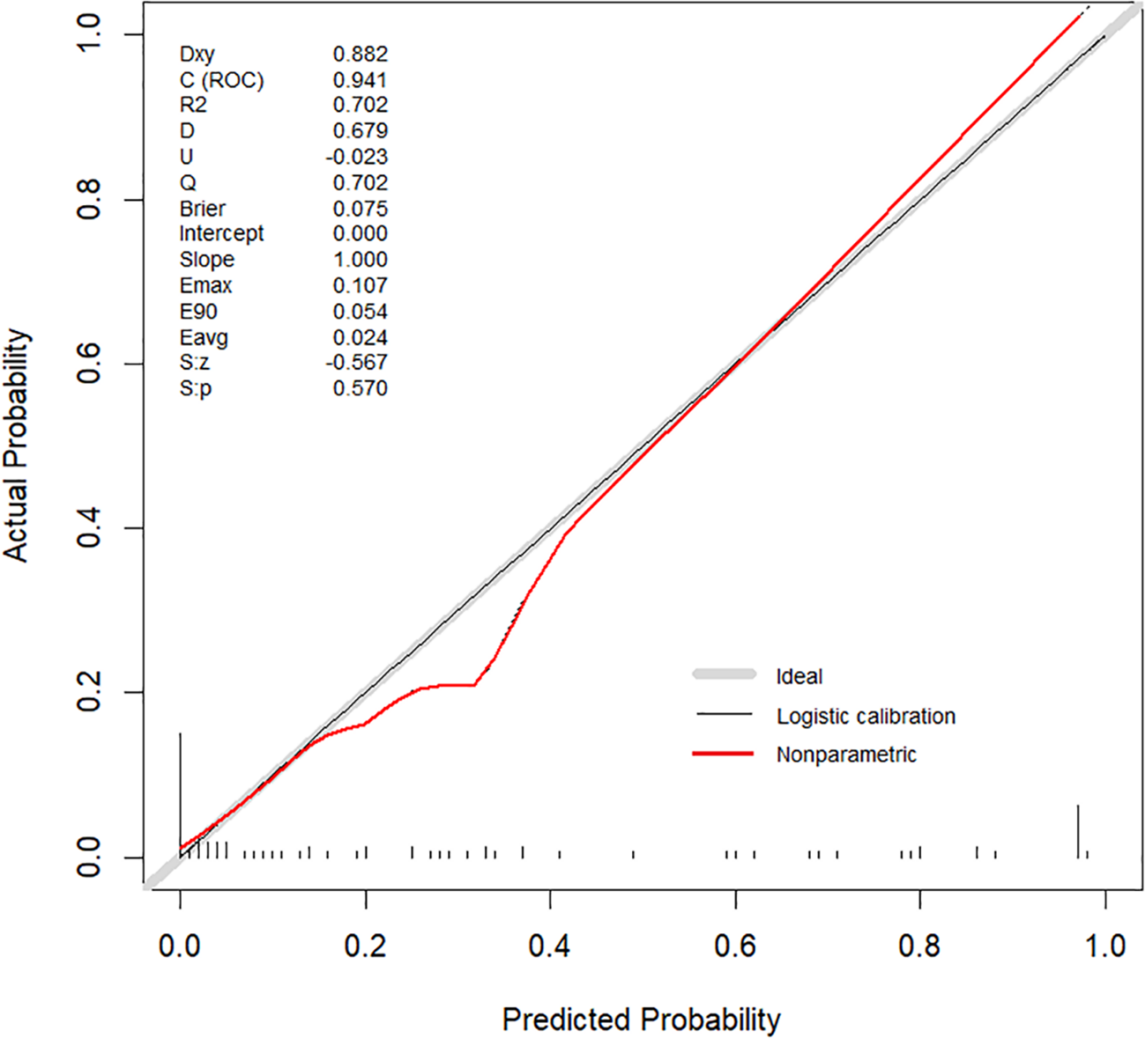
Calibration curve of the MTC nomogram.

**Figure 6:**
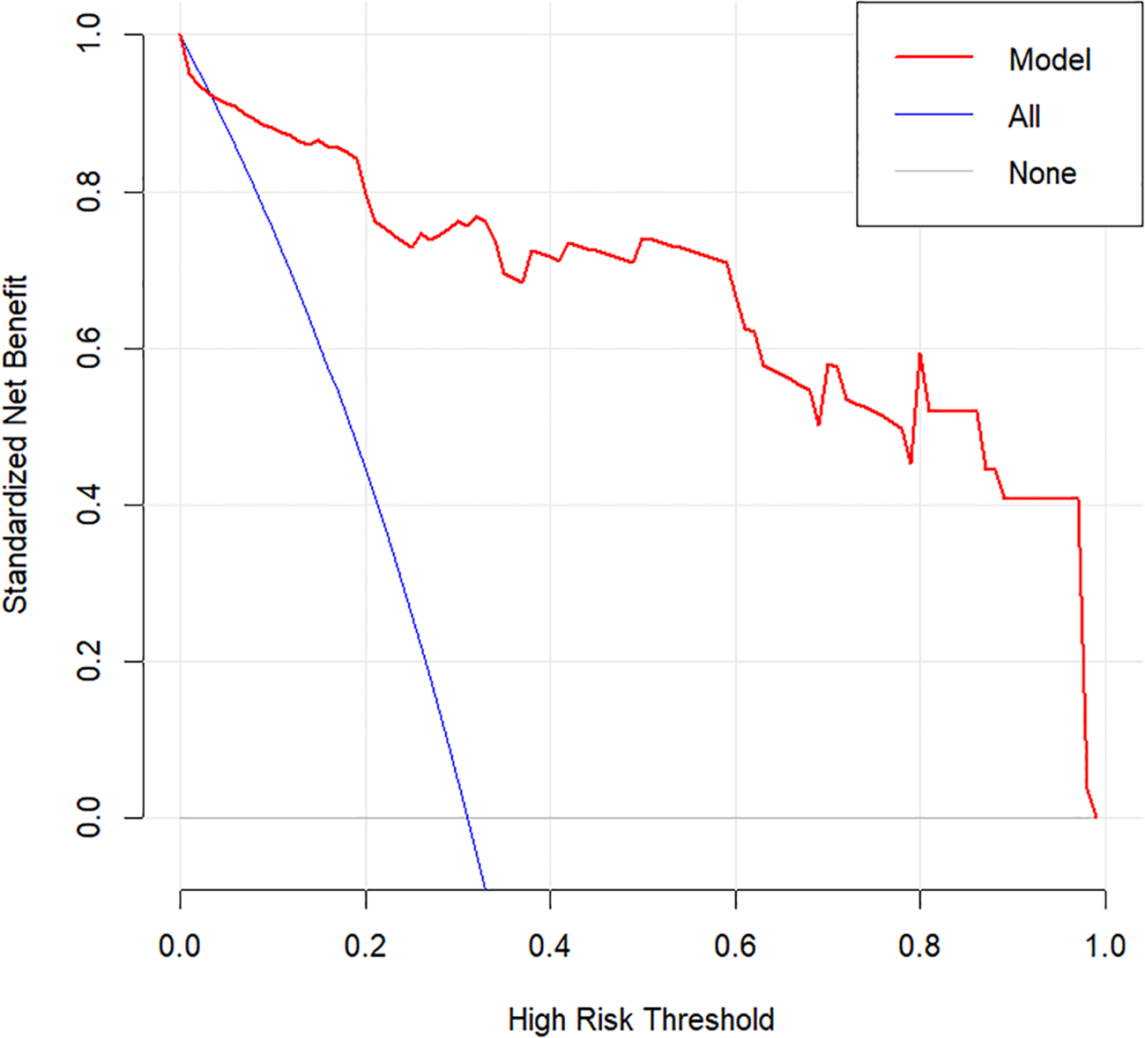
Decision curve analysis of the MTC nomogram.

Internal validation was performed using 1000 bootstrap resamples. The calibration curve (**Figure 5**) closely aligned with the ideal diagonal line, indicating strong agreement between predicted and observed outcomes. The Brier score was 0.075, reflecting high model accuracy. Additionally, the Hosmer–Lemeshow goodness-of-fit test yielded a non-significant result (P = 0.570), confirming good model calibration. DCA further demonstrated the clinical utility of the nomogram across a wide range of threshold probabilities (0.05–1.00), outperforming both the “treat-all” and “treat-none” strategies (**Figure 6**).

## 4 Discussion

MTC presents unique clinical challenges due to its rarity — comprising only 1–2% of all thyroid malignancies, which has hindered establishment of standardized treatment protocols compared with differentiated thyroid cancers. Despite its low incidence, MTC demonstrates a marked propensity for early cervical lymph node metastasis, leading many clinicians to recommend extensive surgical resection including total thyroidectomy with central lymph node dissection and often lateral compartment clearance. While achieving optimal locoregional control, such comprehensive resections carry significant morbidity risks: recurrent laryngeal nerve injury (voice impairment), permanent hypoparathyroidism (chronic hypocalcemia), and cervical sensory disturbances — all substantially impacting quality of life. Importantly, since MTC metastases lack radioiodine avidity, complete surgical excision constitutes the sole potentially curative approach for metastatic disease (15, 16). Consequently, accurate prediction of LLNM is critical in guiding the extent of surgical intervention, balancing oncologic control with the risk of postoperative complications.

In this study, we identified ETE, CLNM, and serum Ctn levels as independent predictors of LLNM. These findings are consistent with previous literature (17–20). Although gender has been linked to LLNM in other reports (21–24), it was not significant in our cohort — likely due to sample size constraints inherent in rare disease research. The nomogram developed from our multivariate analysis integrates three key predictors — ETE, CLNM, and Ln_Ctn — offering a practical tool for individualized LLNM risk estimation. Notably, both serum Ctn and ETE status are preoperatively assessable predictive indicators. While CLNM status is typically confirmed postoperatively through histopathology, it can nevertheless provide valuable preoperative and intraoperative guidance. For instance, preoperative fine-needle aspiration cytology can confirm CLNM, while intraoperative frozen section biopsy of central lymph nodes may serve as a supplementary predictive modality for LLNM assessment.

Ctn, synthesized from procalcitonin, remains the most sensitive biochemical marker for MTC diagnosis (25). The incidence of MTC without elevated Ctn levels is only 0.8% (26, 27). Relevant studies have shown that Ctn levels correlate strongly with both tumor burden and metastatic potential, serving as a reliable prognostic indicator (28, 29). Preoperative Ctn assessment combined with ultrasonography guides clinical decisions regarding LLND (1, 8). However, considerable variability exists in proposed Ctn thresholds for predicting nodal metastasis, with no consensus in current guidelines. Zhu et al. identified 302.5 pg/mL as the Ctn threshold for increased CLNM risk in a single-institution retrospective analysis (30). Park et al. established differential thresholds: 20 pg/mL (ipsilateral LLNM), 200 pg/mL (contralateral LLNM), and 500 pg/mL (distant metastases) through their institutional cohort study (10). It should be noted that these cutoff values have not been validated by multicenter studies or incorporated into clinical guidelines. In our cohort, Ln_Ctn exhibited strong predictive performance for LLNM, achieving an AUC of 0.880 and an optimal cutoff value of 421 pg/mL. These findings confirm Ctn’s robust discriminative capacity for LLNM prediction within our study population, though the generalizability of specific thresholds requires external validation. However, elevated Ctn levels are not exclusive for MTC and may also arise from various benign and pathological conditions, such as autoimmune thyroiditis, hypergastrinemia-related disorders, neuroendocrine neoplasms, chronic renal disease, or pharmacological factors (e.g., prolonged proton pump inhibitor therapy) (31–33), underscoring the need for cautious interpretation and context-specific application of these biochemical thresholds. Regarding the identification of non-MTC related Ctn elevation, we recommend routine preoperative evaluation to exclude secondary causes such as chronic kidney disease, thereby reducing the likelihood of false-positive predictions.

ETE was a particularly strong predictor of LLNM in our model. ETE reflects advanced local invasiveness, indicating tumor penetration through the thyroid capsule - a critical anatomical barrier against metastatic spread. Scopsi et al.’s retrospective cohort study of MTC patients established ETE as a significant prognostic factor for both locoregional recurrence and poor clinical outcomes (34). Our analysis revealed that ETE confers a 13-fold increased risk of LLNM development in MTC patients. These results corroborate prior evidence linking ETE with elevated risks of both lateral compartment involvement and disease recurrence (14, 35).

CLNM serves as a crucial bridge in the metastatic progression of thyroid carcinoma to lateral neck compartments. Although skip metastases (defined as lateral compartment nodal involvement without CLNM) observed in a subset of thyroid carcinoma patients, most metastases progress centrifugally from central to lateral compartments following anatomical lymphatic drainage pathways (36, 37). Machens et al. reported that approximately 70% of MTC patients with CLNM subsequently developed LLNM (38). The United Kingdom National Multidisciplinary Guidelines recommend prophylactic LLND for MTC patients presenting with CLNM (39). In our cohort, CLNM increased the odds of LLNM nearly fivefold. All CLNM cases in this study were pathologically confirmed postoperatively. Although definitive pathological confirmation of CLNM is generally unavailable preoperatively, this nomogram prediction model provides crucial guidance for preoperative and intraoperative prediction of LLNM. Clinically applicable scenarios for the model include: (1) preoperatively confirmed metastasis via central lymph node fine-needle aspiration cytology; (2) intraoperative frozen-section biopsy-confirmed CLNM. We therefore recommend routine implementation of either preoperative central lymph node biopsy or intraoperative frozen-section analysis for MTC patients, as this dual-pathway approach significantly improves therapeutic targeting accuracy.

Nomograms offer an intuitive and individualized approach to risk prediction, particularly in oncology, where complex and heterogeneous variables interact (40). Our study developed a nomogram incorporating three clinically accessible parameters (ETE, CLNM, and Ctn) representing imaging, pathological, and serological dimensions of MTC evaluation. While the model demonstrated satisfactory discrimination, calibration, and net benefit in our cohort, we acknowledge that these findings require cautious interpretation given the study’s retrospective design, single-center nature, and moderate sample size. we acknowledge the potential for false-negative results in clinical practice. To mitigate this risk, we recommend: (1) close postoperative monitoring with serial calcitonin testing and US surveillance, particularly during the first 2 years; (2) intraoperative frozen section examination of suspicious lymph nodes regardless of nomogram prediction; and (3) individualized management for cases with discordant clinical and model findings.

The proposed risk-stratified surgical approach — where patients with higher nomogram scores might benefit from lateral neck dissection while those with lower scores could potentially undergo central compartment dissection alone — represents a hypothesis-generating framework rather than definitive clinical guidance. This tool may eventually help balance oncologic control against surgical morbidity, but its current value lies primarily in highlighting the need for better preoperative risk stratification in MTC management.

Importantly, several limitations warrant consideration: (1) potential referral bias at our tertiary center, (2) variability in preoperative imaging interpretation, (3) absence of molecular or radiomic markers in the current model, and (4) lack of external validation. Future multicenter studies with standardized imaging protocols and incorporation of emerging biomarkers are needed to validate these preliminary findings before clinical implementation.

## 5 Conclusion

This study identified ETE, CLNM, and serum Ctn levels as independent predictors of LLNM in patients with MTC. Based on these variables, we developed a novel nomogram that demonstrated high discriminative accuracy and clinical utility. By enabling personalized risk stratification, this tool may support more precise surgical decision-making — maximizing oncologic control while minimizing unnecessary morbidity. Prospective multicenter validation is warranted to confirm its generalizability and facilitate its integration into clinical practice.

## Data Availability

The original contributions presented in the study are included in the article/supplementary material. Further inquiries can be directed to the corresponding author.
